# Supplementary material for: Dinosaur origin of egg color: oviraptors laid blue-green eggs
Source: PeerJ. 2017 Aug 29;5:e3706. doi: 10.7717/peerj.3706 (PMC5580385; doi:10.7717/peerj.3706)
Supplement: Supplemental Information 1 — Detailed descriptions of the applied methodology and further material information. [file peerj-05-3706-s001.docx]

Supplemental Information

1. **Materials and methods**
2. Institutional abbreviations
3. Description of eggshell specimens
4. Solvents and equipment
5. Extraction and pigment detection
6. Porosity measurements and water vapor conductance estimations
7. Microstructural evidence for cuticle preservation
8. **Supplemental References**
9. **Materials and methods**
10. **Institutional abbreviations**

NMNS: National Museum of Natural Sciences, Taichung, Taiwan

STIPB: Steinmann Institute of Geology, Mineralogy, and Paleontology, Division of Paleontology, University of Bonn, Bonn, Germany

ZFMK: Zoologisches Forschungsinstitut und Museum Alexander Koenig, Bonn, Germany

1. **Description of eggshell specimens**

Extant eggshell material:

The emu (*Dromaius novahollandiae*) eggshells were from captive birds and stored in the ZFMK collections (ZFMK uncat.).

Fossil eggshell material:

Elongatoolithidae Zhao, 1975

*Macroolithus* Zhao, 1975

*Macroolithus yaotunensis* Zhao, 1975

(Laid by the oviraptorid *Heyuannia huangi*)

We obtained several *Macroolithus yaotunensis* eggshell samples from the collections of the NMNS and the STIPB for chemical analysis and porosity measurement. The specimens were collected from three different localities in China, including the Liquangqiao Basin in Henan Province, the Hongcheng Basin in Jiangxi Province, and the Nanxiong Basin in Guangdong Province. The geological background is discussed in detail below.

The eggshells collected from Upper Cretaceous Hugang Formation of the Liguanqiao Basin near Nanyang, southwestern Henan Province, China, have been housed in STIPB since 1985 and were described in Erben (1995). These eggshells are assigned to *Macroolithus yaotunensis* based on their linearituberculate ornamentation, angusticanaliculate pores, and shell thickness.

The oviraptorid eggshells collected from the Nanxiong Basin, located in the northern part of Guangdong Province, China, can also be assigned to *Macroolithus yaotunensis* based on their macro- and microstructure. The NE-SW striking basin has produced numerous theropod egg clutches and eggshell fragments. The Late Cretaceous strata of the basin are divided into the Yuanpu Formation and the overlying Pingling Formation. The Yuanpu Formation was dated at 67 Ma (Zhao et al., 1990).

The *Macroolithus yaotunensis* eggshells from the Hongcheng Basin, southern Jiangxi Province, China, were used for porosity measurement in addition to the chemical analysis. The eggshell samples for chemical analysis were taken from CYN-2004-DINO-05/I (Fig. 1A, left egg) housed in the NMNS and identified as *Macroolithus yaotunensis*. The egg is one of a pair (Fig. 1A) which was excavated from the Upper Cretaceous Nanxiong Formation of the Hongcheng area near Ganzhou City. The sediment in which the two eggs are embedded mainly consisted of tan-red terrestrial sandstone. This sediment was analyzed as control. Four pieces of eggshell were also taken from the blunt, middle, and acute parts of the left egg for measuring the porosity and calculating the water vapor conductance (see section below). The eggs are ornamented with linearituberculate ridges along their long axis, which were considered as the possible pathway for gas exchange (Hirsch & Quinn, 1990; Sabath et al., 1991).

In summary, all eggshells can confidently be referred to *Macroolithus yaotunensis* Zhao (1975) based on their identical micro- and macrostructure. In addition, they can confidently be assigned to the oviraptorid *Heyuannia huangi* because on the identity of their micro- and macrostructure with that of the eggshells of the paired eggs inside a female oviraptorid pelvis from one of these localities, the Hongcheng Basin, Jiangxi Province and with embryo-bearing eggs of the same taxon (Sato et al., 2005).

1. **Solvents and equipment**

In our analyses, we used protoporphyrin IXα and biliverdin hydrochloride as commercial standards and purchased these from Sigma Aldrich, UK. Disodium EDTA was obtained from Merck, Germany. Acetic acid (100%, LC-grade) and acetonitrile (LC-MS-grade) were also purchased from Merck, Germany, and from Sigma Aldrich, UK.

For extraction of the eggshell color pigments and filtration of the extract, we used 1.5 mL light-protecting polypropylene Eppendorf Amber microcentrifuge tubes and 0.5 mL standard conical Eppendorf microcentrifuge tubes. For vortex-mixing, we used a vortex mixer K neoLab (7-2020) and for centrifuging an Eppendorf centrifuge. Before injecting the eggshell extracts into the mass spectrometer, we filtered them by using a Chromafil O-45/3 PTFE disposable syringe filter holder (pore diameter: 0.45µm, filter diameter: 3 mm) on a 1.00 mL Injekt-F sterile syringe with a Sterican cannula on top.

Mass spectra were recorded on a micrOTOF-Q mass spectrometer (Bruker) with an ESI-source coupled with an HPLC Dionex Ultimate 3000 instrument (Thermo Scientific) using an EC50/2 Nucleodur C18 Gravity 3 µm column (Macherey-Nagel).

1. **Extraction and pigment detection**

The eggshell samples we used for pigment analysis were separately stored in air-tight plastic ziplets, which were protected from light in opaque paper boxes. We used eggshell fragments of at least 0.036 cm^2^ surface area, weighing between 184 mg and 562 mg (Table S1). These eggshell fragments were cleaned from attached sediment with deionized, distilled water and then broken into smaller pieces.

The following extraction procedure is based on the method established by Gorchein et al. 2009.

Due to the greater polarity of BV as compared to PP, BV is partially dissolved in the aqueous EDTA solution; this results in a significant loss of BV during the extraction process. To quantify the amount of BV dissolved in the EDTA, the EDTA supernatants were stored for later UV/Vis measurements. The decalcification routine was repeated three times for BV and PP extraction.

Reverse-phase HPLC was run at a flow rate of 0.3 mL/min. HPLC was started with 90% H_2_O containing 0.1% acetic acid. The gradient started after 1 min and reached 100% acetonitrile after 14 min. For an additional 7 min, the column was flushed with 100% acetonitrile (containing 0.1% acetic acid). For the BV analysis,15 µL sample solution were injected, and 20 µL for the PP detection.

Data were collected in positive full scan MS mode over the range of 50 – 1000 m/z, using a capillary voltage of 4.5 kV and an end plate offset of -500 V. The dry heater of the ESI source was set at 200°C. Nitrogen desolvation and nebulizer gas flow was 10.0 L/min, the nebulizer was run at 2.2 bar. Time-Of-Flight (TOF) detection allowed the determination of the accurate masses of BV and PP.

The observed elution order was as follows: BV (after 8 min; double peak indicates at least two isomers in acetonitrile-acetic acid) and PP (after 14 min; double peak indicates at least two isomers in acetonitrile-acetic acid).

1. Empirical relationships for estimating preserved BV concentration:

${\text{c}_{\text{BV}}^{\text{tot}}\text{ ≥ c}}_{\text{BV}}\text{ × }\text{m}_{\text{X1g}}\text{ × }\text{f}_{\text{ex}}\text{ × }\text{f}_{\text{c}}$ (1)

$$\text{c}_{\text{BV}}^{\text{tot}}\text{=preserved total amount of BV stored into 1 g of eggshell }\left[ \frac{\text{nmol}}{\text{1g}\left( \text{eggshell} \right)} \right]$$

$$\text{c}_{\text{BV}}\text{=ESI MS concentration approximation }\left[ \frac{\text{nmol}}{\text{Xg}\text{ (mass eggshell fragment)}} \right]$$

$$\text{m}_{\text{X1g}}\text{=mass multiplication factor for each eggshell fragment mass to reach 1g }$$

$$\text{f}_{\text{ex}}\text{=const. }\left( \text{for our extraction routine} \right)\text{≈3}$$

$$\text{f}_{\text{c}}\text{=const. }\left( \text{for empirical correction} \right)\text{≈4}$$

$$\text{→12 × }\text{c}_{\text{BV}}\text{ × }\text{m}_{\text{X1g}}$$

1. Empirical relationships for estimating preserved PP concentration:

${\text{c}_{\text{PP}}^{\text{tot}}\text{ ≥ c}}_{\text{PP}}\text{ × }\text{m}_{\text{X1g}}\text{ × }\text{f}_{\text{ex}}\text{ × }\text{f}_{\text{c}}$ (2)

$$\text{c}_{\text{PP}}^{\text{tot}}\text{=preserved total amount of PP stored into 1 g of eggshell}\left[ \frac{\text{nmol}}{\text{1g}\left( \text{eggshell} \right)} \right]$$

$$\text{c}_{\text{PP}}\text{=ESI MS concentration approximation }\left[ \frac{\text{nmol}}{\text{Xg}\text{(mass of each eggshell fragment)}} \right]$$

$$\text{m}_{\text{X1g}}\text{=multiplication factor for each eggshell fragment mass to reach 1g }$$

$$\text{f}_{\text{ex}}\text{=const. }\left( \text{for our extraction routine} \right)\text{≈1}$$

$$\text{f}_{\text{c}}\text{=const. }\left( \text{for empirical correction} \right)\text{≈4}$$

$$\text{→4 × }\text{c}_{\text{PP}}\text{ × }\text{m}_{\text{X1g}}$$

**Table S1** Estimation of the preserved pigment amounts in the analyzed eggshell samples.


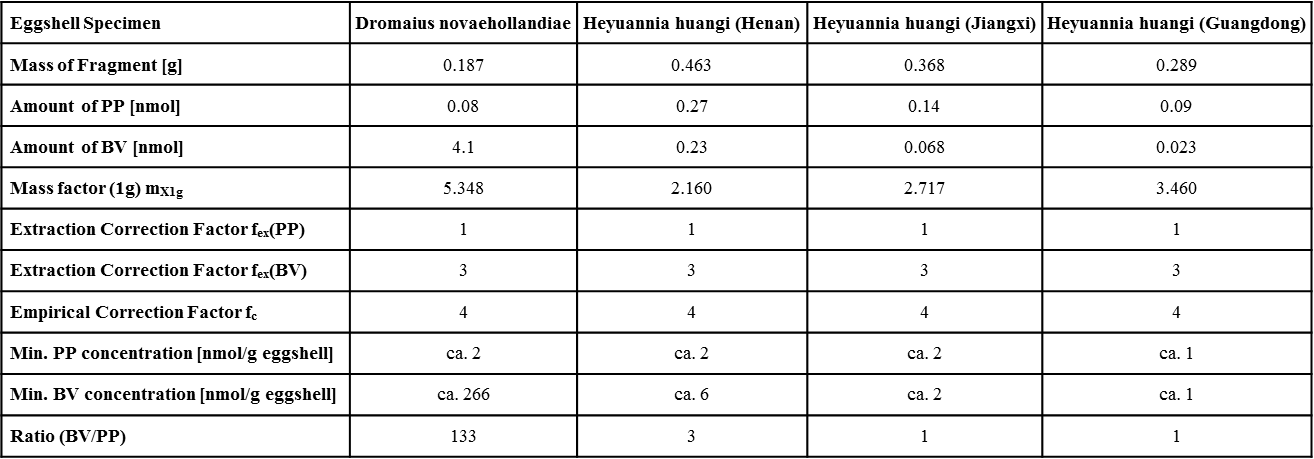


1. **Porosity measurement and water vapor conductance estimation**

We measured eggshell porosity in four eggshell tangential sections of *Heyuannia* egg CYN-2004-DINO-05/I (Fig. 1A, right egg) under the polarized light microscope using a modification of the zonal division approach (zones 1 to 4, Z_1_ to Z_4_ in the equation) and asymmetrical egg model applied to troodontid eggs (Varricchio et al., 1997). To take the asymmetrical shape of the oviraptorid egg with its pointed and blunt ends into account, we modelled the shape of the egg as two half prolate ellipsoids and two cones (Fig. 1B). The blunt end (Z_1_) and the acute end (Z_4_) were modeled as half prolate ellipsoids with a radius of 4.05 cm and 2.65 cm, respectively, and long axes of equal length (5.7 cm). The rest of the egg between the poles was further divided into two zones (Z_2_ and Z_3_) which were modelled as two cones (cone height = 4.3 cm and cone radius 4.05 cm and 2.65 cm, respectively). The geometric equations used for calculating egg volume and surface area are given in Table S2, as are the regression equations for modern bird eggs that we used for calculating volume, surface area and water vapor conductance of egg CYN-2004-DINO-05/I. The geometric parameters of the model are provided in Table S4.

The areas of all individual pores in each tangential section were summed and compared with the total surface area of each zone to calculate the percentage of pore area. This percentage was assumed to apply uniformly to the entire zone and used to calculate the total pore area per zone. Next, the equation (Eq. 3) proposed by Jackson et al. (2008) was used to calculate water vapor conductance of the *Heyuannia* egg by zone and overall conductance.

$\text{G}_{\text{H}_{\text{2}}\text{O}}\text{ =}\left( \frac{\text{c}}{\text{RT}} \right)\text{× }\text{D}_{\text{H}_{\text{2}}\text{O}}\text{×(}\text{A}_{\text{p}}\text{/}\text{L}_{\text{s}}\text{)}$ (3)

A_p_ and L_s_ are the total pore area and the average pore length, respectively. R represents the universal gas constant which is 6.236×10^4^ cm^3^ Torr mol^-1^ K^-1^. T is the temperature in K. c is a conversion factor changing both seconds to days and moles to milligrams of water. c is 1.5552*10^9^ sec mg H_2_O day^-1^ mol^-^1. The binary diffusion coefficient of water vapor in air changes with temperature and is 0.292 cm^2^ s^-1^ at 30°C. Pore canal length was assumed to be equal to eggshell thickness which is constant for each zone but varies between zones. Accordingly, pore canal length differs in different zones.

The total volume of egg CYN-2004-DINO-05/I is 260.3 cm^2^. The egg’s weight was estimated as 276 g by applying an average avian egg density of 1.06 g/cm^3^ (Deeming, 2006). The $\text{G}_{\text{H}_{\text{2}}\text{O}}$ of an avian egg of equivalent weight is 99.3 mg H_2_O day^-1^ Torr^-1^ according to the regression equation proposed by Jackson et al. (2008). This value is slightly lower than our result (108.66 mg H_2_O day^-1^ Torr^-1^) (the sum of the values for the four zones in Table S3). We compared our results with those published by Mou (1992), which is 231 mg H_2_O day^-1^ Torr^-1^ and thus significantly higher than our results. Mous's results were also cited by Deeming (2006). The higher values of Mou (1992) result from his assumption that porosity is homogeneously distributed over the entire egg; however, this assumption proved incorrect for elongated eggs (Varricchio et al., 1997). In the Mou’s study, several pieces from the middle part of egg were taken for porosity estimation without considering the heterogeneous porosity distribution. Our results (Table S4) show that the highest porosity is found in the middle zones, especially in zone 3, which is mainly covered by linearituberculate ornamentation. Hence, the results of Mou (1992) overestimate water vapour conductance mainly because he extrapolated the porosity of the middle part of the egg to the entire egg.

1. **Microstructural evidence for cuticle preservation**

In extant bird eggs, the cuticle layer is composed of an inner calcified layer and an outer non-calcified layer. The inner calcified layer consists of a thin layer of hydroxyapatite (Dennis et al., 1996). Eggshell from an embryo-bearing *Heyuannia* egg (NMNS-0015726-F02-embryo-01) was radially sectioned for observation under a polarized light microscope. In the radial section of this eggshell specimen, we observed a mineralized layer with mammillae-like structures atop the crystalline layer of the eggshell, which may be the preserved inner hydroxyapatite layer of the original cuticle (Fig. S2). This observation is consistent with the detection of PP, because PP is mainly deposited in the cuticle.

**Table S2** Geometric equations used for calculating volume and surface area of the fossil egg and regression equations for modern bird eggs used for calculating volume, surface area and water vapor conductance of the fossil egg.

| **Geometric equations** | |
| --- | --- |
| Half prolate ellipsoid (for zones 1 and 4) | V = $\frac{\text{2}}{\text{3}}\text{π}\text{abc}$, for prolate ellipsoid, a=b=4.05 cm (blunt end) or 2.65 cm (acute end).  S =$\text{2π}\left( \text{a}^{\text{2}}\text{+}\text{c}^{\text{2}}\text{× }\frac{\text{∈}}{\tan\text{∈}} \right)$, $\text{∈}\text{=}\text{cos}^{\text{-1}} \frac{\text{a}}{\text{c}}\text{ (}\text{eccentricity}$) |
| Cone  (for zones 2 and 3) | V = $\frac{\text{1}}{\text{3}}\text{π}\text{r}^{\text{2}}\text{h}$, r: the radius of the bottom circle.  S = $\text{π}\text{r}{\text{(}\text{r}^{\text{2}}\text{+}\text{h}^{\text{2}}\text{)}}^{\frac{\text{1}}{\text{2}}}$ (Weisstein, 2011)  y = 6.14x－24.87 (slope of specific cone used in this model). |
| **Regression equations** | |
| Egg volume | V_egg_ = 0.51*LB* ^2^, *L* ≡ egg length, *B* ≡ egg breadth (Hoyt, 1979). |
| Egg surface area | S_egg_ = 4.951 *V* ^0.666^ (Paganelli et al., 1974). |
| Egg conductance | $G_{H_{2}O}$ = 0.3786*M* ^0.818^, M ≡ egg mass (Jackson et al., 2008). |

**Table S3** Zonal dimensions of the modelled *Heyuannia* egg CYN-2004-DINO-05/I (Fig. 1A, right egg).

| **Zone** | **Height (cm)** | **% Total egg height** | **Volume (cm^3^)** | **% Volume** | **Surface area**  **(cm^2^)** | **% Surface area** | **Pore canal length (mm)** |
| --- | --- | --- | --- | --- | --- | --- | --- |
| 1 | 5.7 | 28.5 | 97.9 | 37.6 | 131.95 | 33.3 | 1.6 |
| 2 | 4.3 | 21.5 | 66.6 | 25.6 | 101.28 | 25.6 | 1.5 |
| 3 | 4.3 | 21.5 | 54.0 | 20.7 | 82.12 | 20.7 | 1.5 |
| 4 | 5.7 | 28.5 | 41.9 | 16.1 | 80.62 | 20.4 | 1.6 |

**Table S4** Results of the porosity measurements and calculations of water vapor conductance of the modelled *Heyuannia* egg CYN-2004-DINO-05/I (Fig. 1a, right egg).

| **Zone** | **Total area (cm^2^)** | **Area examined (cm^2^)** | **No. of pores observed** | **Pore diameter (μm)** | **Pore density (no./mm^2^)** | **% Pore area** | **Pore area, Ap (mm^2^)** | $\mathbf{G}_{\mathbf{H}_{\mathbf{2}}\mathbf{O}}$ **(mg/dayTorr)** | **Pores per zone** | **per cm^2^ in zone** |
| --- | --- | --- | --- | --- | --- | --- | --- | --- | --- | --- |
| 1 | 131.95 | 102 | 7 | 56.5 | 0.069 | 0.068 | 9.015 | 13.77 | 906 | 0.104 |
| 2 | 101.28 | 150 | 28 | 57.4 | 0.187 | 0.194 | 19.614 | 31.95 | 1891 | 0.315 |
| 3 | 82.12 | 342 | 108 | 57.2 | 0.316 | 0.326 | 26.789 | 43.64 | 2593 | 0.531 |
| 4 | 80.62 | 107 | 17 | 58.3 | 0.159 | 0.170 | 13.668 | 20.88 | 1281 | 0.259 |

**A**  **B**

**Figure S1** Structural formulas of eggshell pigments (A) BV and (B) PP.


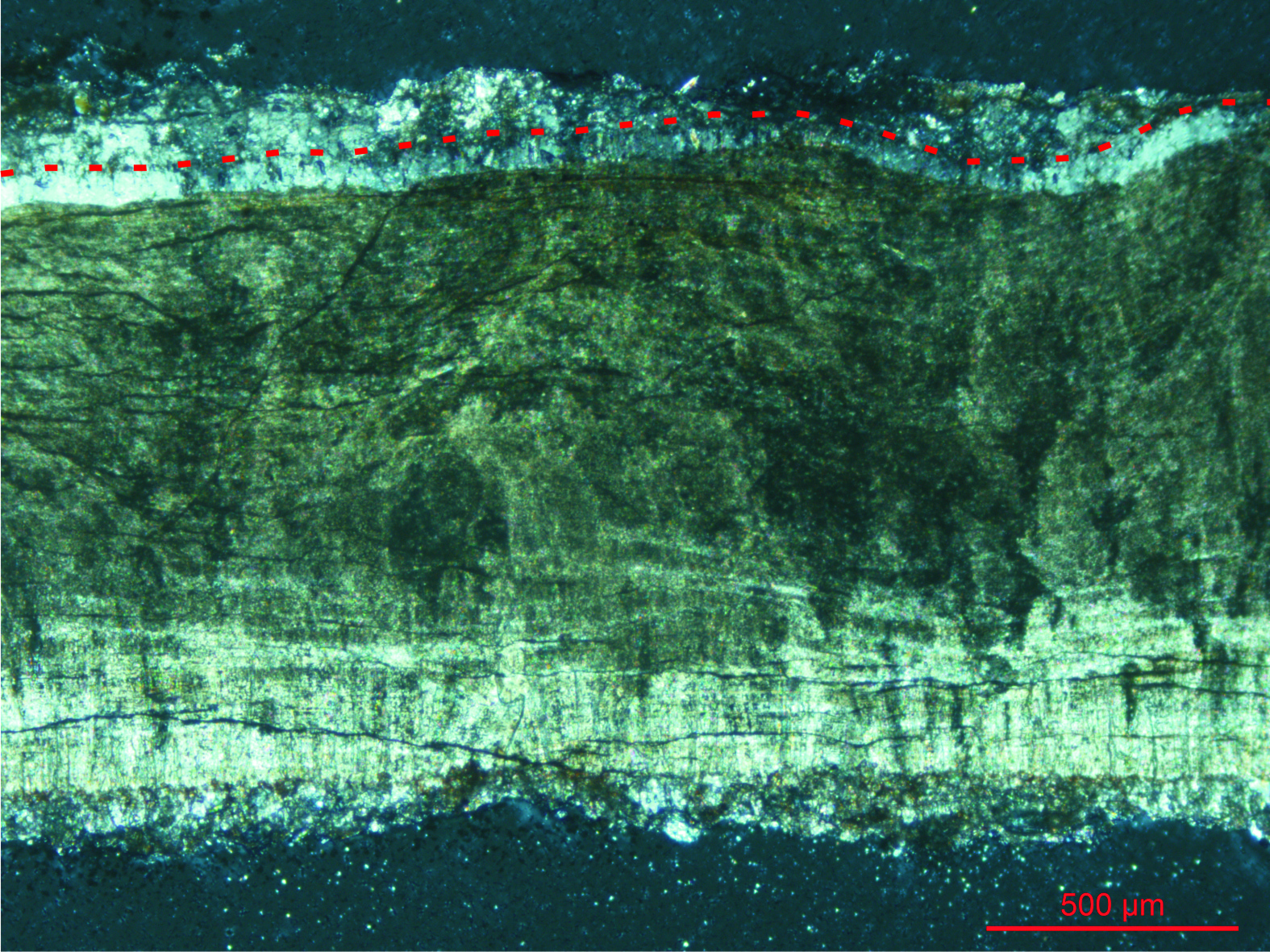


**Figure S2:** Radial section of eggshell from an embryo-bearing *Heyuannia* egg (NMNS-0015726-F02-embryo-01) in polarized light showing the putative cuticle layer below the red dashed line and the outer surface of the brownish crystalline layer. Scale bar equals 500 μm.

2. Supplemental References

Zhao, Z.-Q. Microstructures of the dinosaurian eggshells of Nanxiong, Guangdong, and the problems in egg classification. *Vertebrata Palasiatica* 13, 105-117 (1975) (in Chinese with English abstract).

Erben, K. H. *The Cretaceous/Tertiary boundary in the Nanxiong-Basin (continental facies, SE-China)*. (Franz Steiner Verlag, 1995) (in German)

Zhao, Z.-Q., Ye, J., Li, H.-M., Zhao, Z.-H. & Yan, Z. Extinction of the dinosaurs across the Cretaceous Tertiary boundary in Nanxiong Basin Guangdong Province, *Vertebrata Palasiatica* 29, 1-20 (1991) (in Chinese with English abstract).

Hirsch, K. F. & Quinn, B. Eggs and eggshell fragments from the Upper Cretaceous Two Medicine Formation of Montana. *Journal of Vertebrate Paleontology* 10, 491-511 (1990).

Sabath, K. Upper Cretaceous amniotic eggs from the Gobi Desert. *Acta Paleontologica Polonica* 36, 151-192 (1991).

Sato, T., Cheng, Y.-N., Wu, X.-C., Zelenitsky, D. K. & Xiao, Y.-F. A pair of shelled eggs inside a female dinosaur. *Science* 308, 375 (2005).

Jackson, F. D., Varricchio, D. J., R. A. Jackson, Vila, B. & Chiappe, L. Water vapour conductance of a titanosaur egg (*Megaloolithus patagonicus*) from Argentina: comparison with a *Megaloolithus siruguei* egg from Spain. *Paleobiology* 34, 229-246 (2008).

Mou, Y. Nest environment of the Late Cretaceous dinosaur eggs from Nanxiong Basin, Guangdong Province. *Vertebrata Palasiatica* 30, 120-134 (1992).

Deeming, D. C. Ultrastructral and functional morphology of eggshells supports the idea that dinosaur eggs were incubated buried in a substrate. *Palaeontology* 49, 171-185 (2006).

Paganelli, C. V., Olszowka A. & Ar A. The avian egg: surface area, volume, and density. *Condor* 76, 319–325 (1974).

Weisstein, E. W. ‘‘Cone.’’ MathWorld—A Wolfram Web Resource. http://mathworld.wolfram.com/ Cone.html (2011).

Hoyt, D. F. Practical methods of estimating volume and fresh weight of bird eggs. *Auk* 96, 73–77 (1979).

Dennis, J. E., Xiao, S. Q., Agarwal, M., Fink, D. J., Heuer, A. H., & Caplan, A. I. Microstructure of matrix and mineral components of eggshells from white leghorn chickens (Gallus gallus). *Journal of Morphology*, *228*(3), 287-306 (1996).
