# Supplementary figures and images for: Dinosaur origin of egg color: oviraptors laid blue-green eggs
Source: PeerJ. 2017 Aug 29;5:e3706. doi: 10.7717/peerj.3706 (PMC5580385; doi:10.7717/peerj.3706)

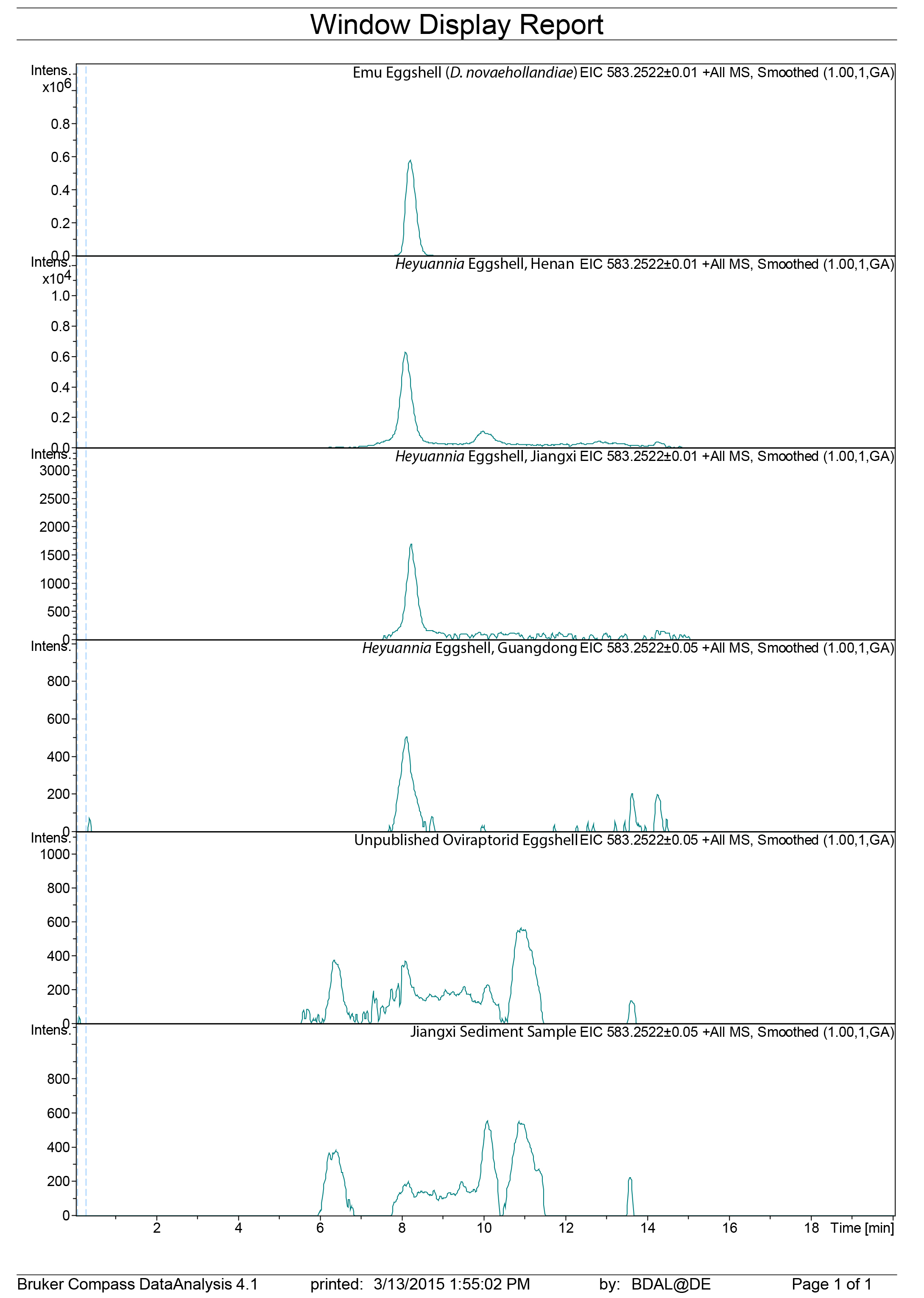

Supplement: Supplemental Information 2 — Raw data extracted ion chromatogram for biliverdin (583 g/mol). [file peerj-05-3706-s002.jpg]

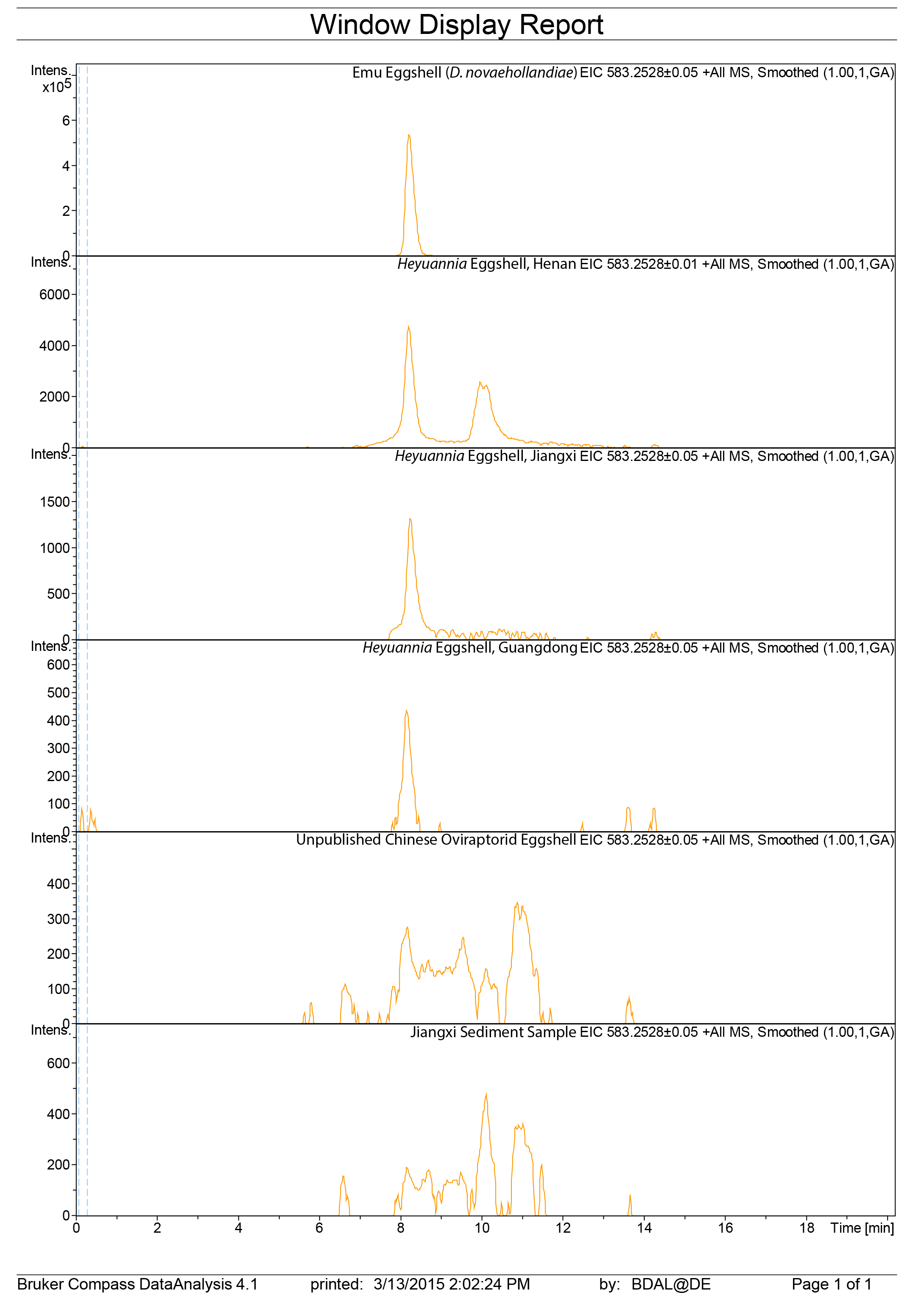

Supplement: Supplemental Information 3 — Raw data of the liquid chromatography mass spectrometry extracted ion chromatograms for biliverdin in a second sample run/repetition. [file peerj-05-3706-s003.jpg]

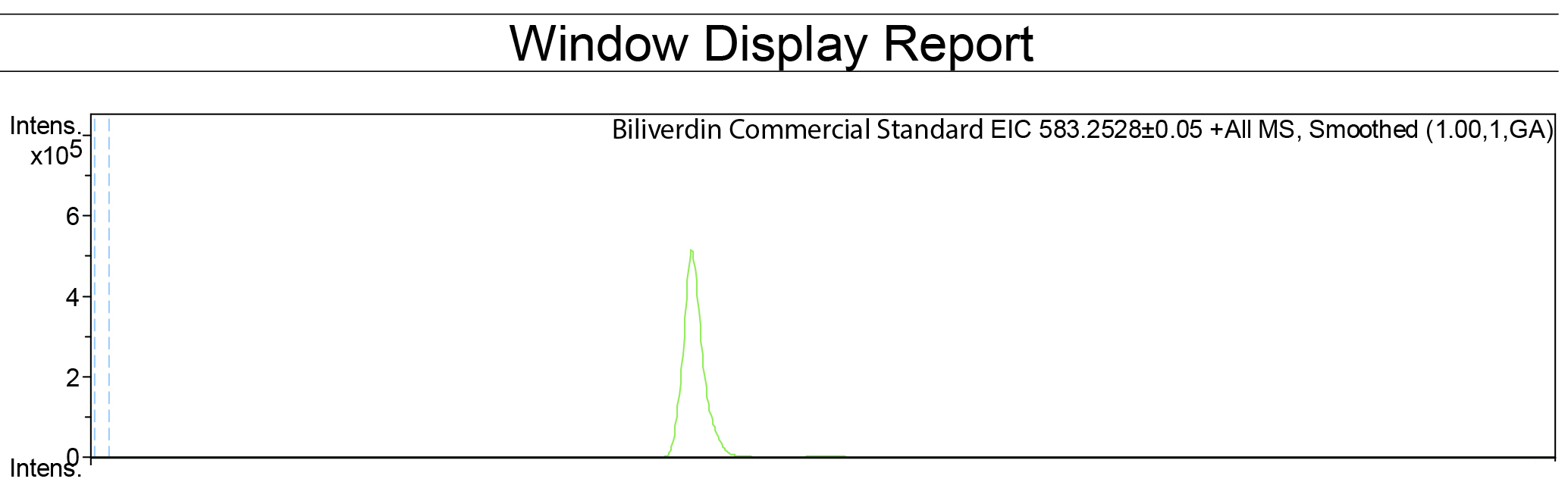

Supplement: Supplemental Information 4 — Extracted ion chromatograms for biliverdin (583 g/mol) of the commercial biliverdin standard. [file peerj-05-3706-s004.jpg]

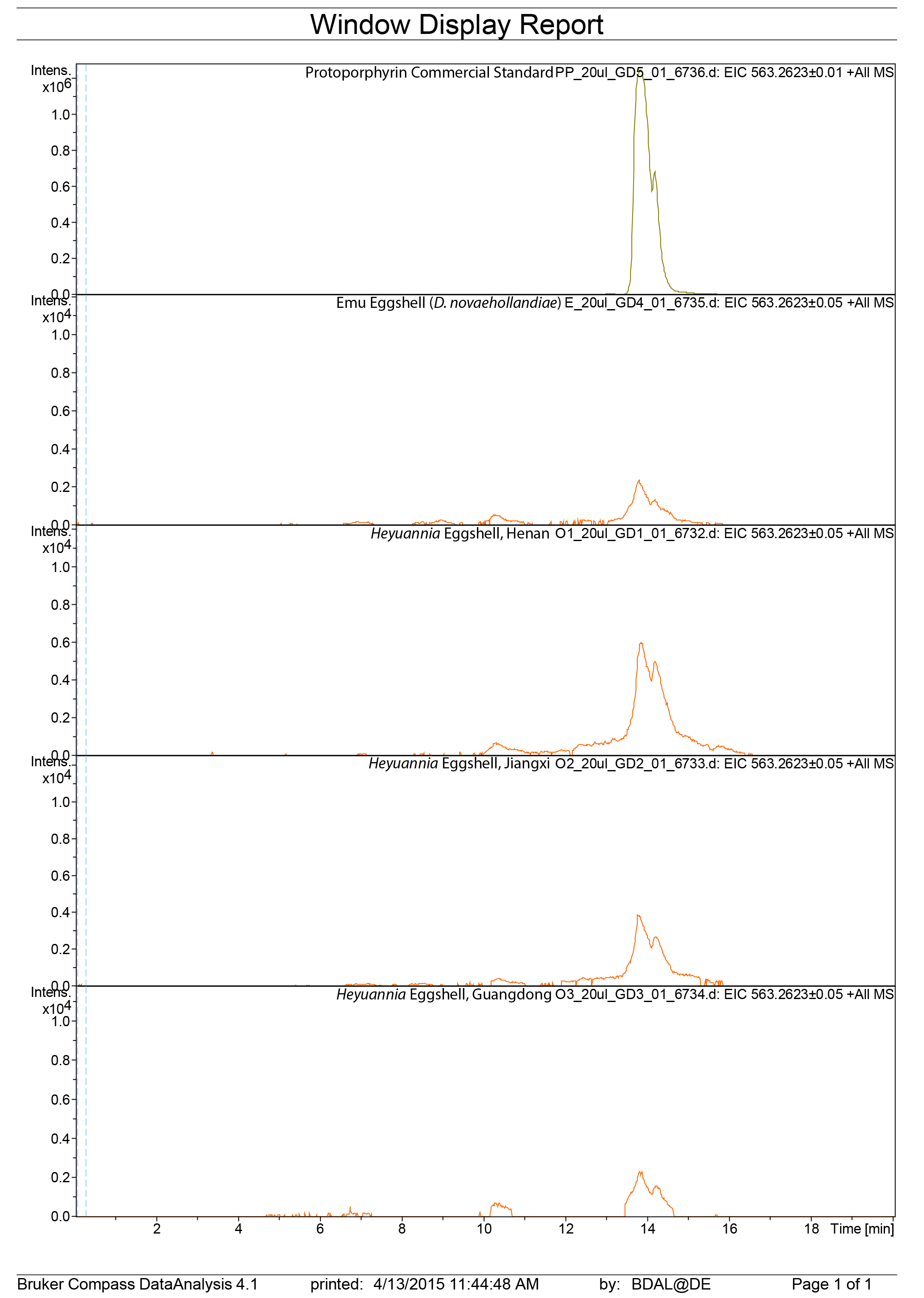

Supplement: Supplemental Information 5 — Sample liquid chromatography mass spectrometry extracted ion chromatograms for protoporphyrin (563 g/mol). [file peerj-05-3706-s005.jpg]

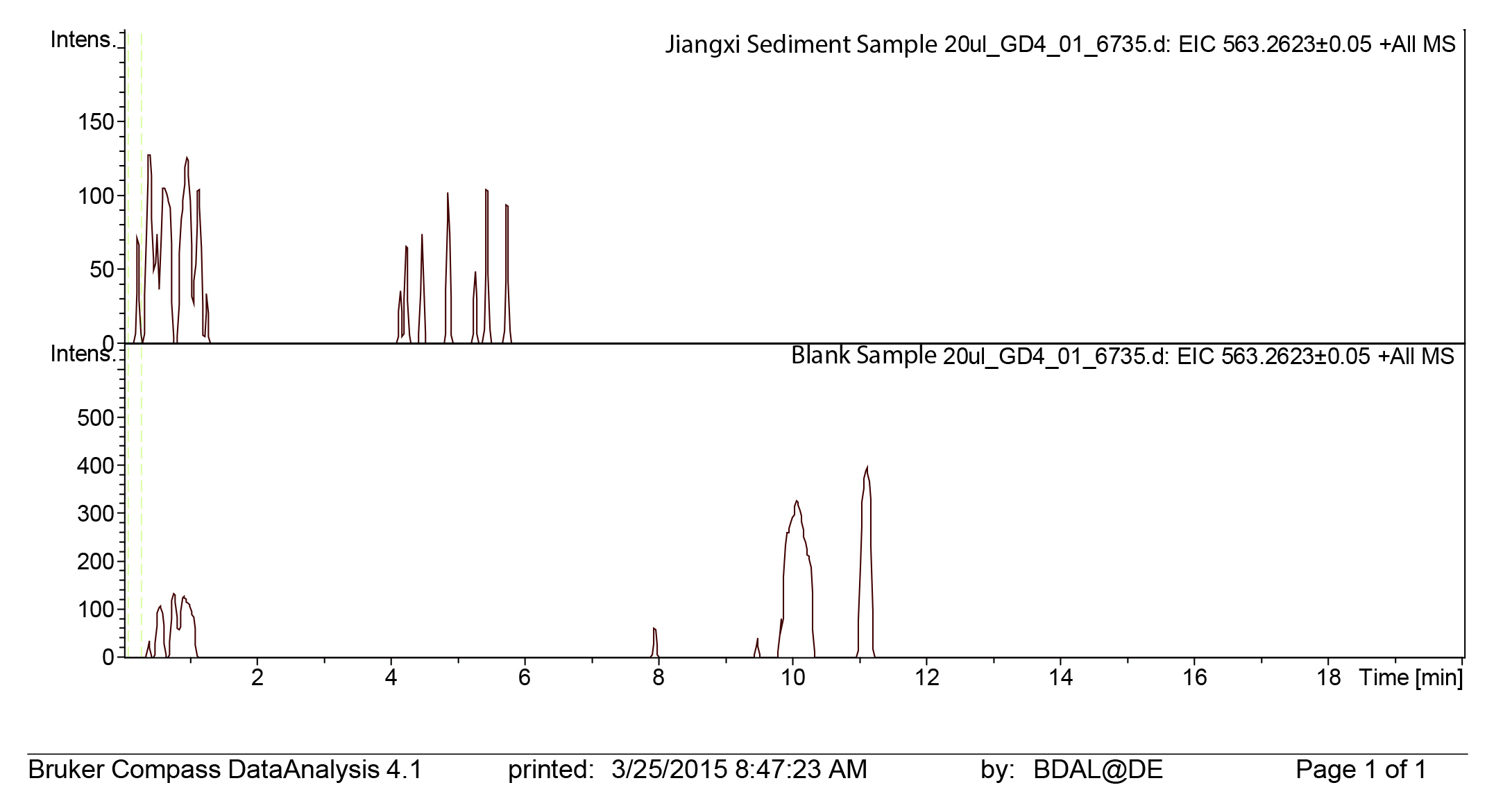

Supplement: Supplemental Information 6 — LC mass spectrometry extracted ion chromatograms for protoporphyrin (563 g/mol) for the Jiangxi sediment sample. [file peerj-05-3706-s006.jpg]
